# Molecular remodelling of Cryptococcus neoformans during adaptation to host temperature

**DOI:** 10.1099/jmm.0.002205

**Published:** 2026-10-07

**Authors:** Marianna Dallastella, Isadora F. Munhoz da Rocha, Bruno A. A. Romagnoli, Lysangela R. Alves

**Affiliations:** 1 Pathogenic Fungi Laboratory Carlos Chagas Institute (Fiocruz Paraná), Curitiba, Brazil; 2 National Institute of Science and Technology in Human Pathogenic Fungi, São Paulo, Brazil

**Keywords:** *Cryptococcus neoformans*, temperature adaptation, transcriptome

## Abstract

**Introduction.** The ability of *Cryptococcus neoformans* to adapt to host temperature is an important feature of its pathogenic potential and requires extensive cellular remodelling.

**Hypothesis/Gap Statement.** Although *C. neoformans* undergoes substantial molecular changes at host temperature, the extent to which transcriptomic and proteomic responses are concordant during this adaptation remains incompletely understood.

**Aim.** We investigated the molecular and metabolic responses of the *C. neoformans* H99 strain grown at 30 and 37 °C using an integrated transcriptomic, proteomic and functional approach.

**Methodology.** RNA sequencing and quantitative proteomics were performed after 24 h of growth at 30 and 37 °C. Differentially expressed transcripts and proteins were identified, and matched transcript–protein features were analysed for directional concordance and quantitative correlation. Cellular metabolic activity was assessed using the 2,3-bis-(2-methoxy-4-nitro-5-sulfophenyl)-2H-tetrazolium-5-carboxanilide (XTT) reduction assay.

**Results.** RNA-seq analysis identified 344 upregulated and 428 downregulated transcripts at 37 °C. Upregulated transcripts were enriched in functions related to ribosome biogenesis, translation, protein folding and mitochondrial-associated processes, whereas downregulated transcripts were associated with carbohydrate metabolism and other metabolic functions. Proteomic analysis revealed a more selective response, with increased abundance of proteins associated with oxidoreductase activity, sterol and isoprenoid biosynthesis, glutamine metabolism and metal-binding functions. Integration of the transcriptomic and proteomic datasets identified 305 matched features, of which 162 (53.1%) showed directional concordance. However, no significant quantitative correlation was observed between transcript and protein fold changes (Spearman ρ = −0.005, *P*=0.934; Pearson r=0.003, *P*=0.959). Cells grown at 37 °C also showed significantly increased XTT reduction, indicating higher cellular metabolic activity under the conditions tested.

**Conclusion.** Adaptation to host temperature involves broad transcriptional remodelling accompanied by selective proteomic changes and increased cellular metabolic activity. These findings provide a molecular framework for further investigating the mechanisms underlying thermoadaptation and their contribution to the pathogenic potential of *C. neoformans*.

## Introduction

The environmental pathogen *Cryptococcus neoformans* is the causative agent of cryptococcosis, a disease that primarily affects immunocompromised individuals and can lead to meningoencephalitis [1]. The transition of the pathogen to the human host relies on multiple adaptive mechanisms, among which thermotolerance is a key determinant [2]. Differential growth of *C. neoformans* across 30, 37, 39 and 41 °C underscores its remarkable thermal adaptability to environmental niches, mammalian hosts and avian reservoirs [3]. Successful adaptation to the human physiological temperature of 37 °C is a prerequisite for host colonization and infection, demanding coordinated metabolic, transcriptional and proteomic remodelling in response to thermal stress.

The impact of temperature on gene expression in *C. neoformans* has been extensively investigated. Early studies compared the transcriptomic profiles of the H99 strain grown under environmental (25 °C) and host-associated (37 °C) temperatures using Serial Analysis of Gene Expression (SAGE) [4], genomic DNA microarrays [5] and representational difference analysis [6]. These studies revealed substantial transcriptional remodelling in response to temperature shifts, highlighting the importance of thermal adaptation for fungal survival and pathogenicity.

Subsequent investigations employing similar experimental approaches identified additional temperature-responsive genes involved in diverse biological processes. Several genes associated with thermotolerance and cellular responses to heat and oxidative stress, including *CNA1* [7], *CNB1* [8], *MPK1* [9], *SOD2* [10] and *TSA1* [11], were found to be differentially expressed under elevated temperatures. Temperature-dependent regulation was also observed for *CCN1* [12], a gene involved in RNA processing, mRNA splicing and spliceosome assembly. In addition, differential expression of *VPH1* [13] and *ILV2* [14], which are associated with capsule organization and melanin biosynthesis, respectively, has been reported. Other temperature-responsive genes include *SPE3/LYS9* [15], implicated in oxidoreductase activity, and *CTS1* [9], which is involved in mating and cytokinesis.

Collectively, these studies demonstrate that adaptation to elevated temperatures requires extensive transcriptional reprogramming affecting stress response pathways, cellular metabolism, virulence-associated traits and fundamental biological processes. Notably, one of the earliest transcriptomic analyses conducted by Steen *et al*. [4, 15] using SAGE revealed increased expression of histone-encoding transcripts at lower temperatures, whereas transcripts associated with the translational machinery and heat shock proteins were enriched under host-temperature conditions.

In the present study, we revisited and expanded upon these pioneering observations by investigating the molecular responses of the reference strain *C. neoformans* H99 during the transition from an environmental to a host-associated temperature. To achieve this, we performed integrated transcriptomic and proteomic analyses following cultivation at 30 and 37 °C for 24 h and compared the responses across both molecular layers. In addition, metabolic activity was quantified to provide a functional assessment of the physiological adaptations associated with temperature-induced stress. This multi-omics approach enabled a comprehensive evaluation of the regulatory mechanisms underlying thermal adaptation in *C. neoformans*.

## Methods

### Cell culture

*C. neoformans* H99 strain (serotype A, wild-type) cells were cultured in agar yeast extract peptone dextrose (YPD) medium or liquid YPD medium under agitation at 150 r.p.m. The cells were grown at 30 or 37 °C.

### Transcriptome

#### RNA extraction

Inocula were standardized at 1×10^8^ cells ml^−1^. Cells were lysed with the addition of glass beads (Sigma – acid-washed glass beads 425–600 µm) and vortexed. Trizol and chloroform were added, and the supernatant was separated and washed with 2-propanol and 75% ethanol. Extraction quality was verified on a Bioanalyzer (Agilent Technologies). The samples were prepared in biological triplicate.

#### RNA library

The sequencing library was constructed with the TruSeq RNA Library Prep Kit v2 (Illumina) according to the manufacturer’s instructions, and all samples were prepared in independent triplicates. RNA-seq was performed on the Illumina MiSeq platform at the Carlos Chagas Institute (Fiocruz – Paraná), using a MiSeq Reagent Kit v2 cartridge.

#### RNA analysis

The sequences were analysed by CLC Genomics Workbench^©^ v 23.0 (Qiagen) in fastq format, using the *C. neoformans* genome corresponding to the H99 strain (ref seq assembly GCF_000149245.1). The parameters used for the alignments were: cost per base exchange (2), insertion cost (3), deletion cost (3), length fraction (0.7) and similarity fraction (0.8). Reads mapped 30 times along the genome were considered in the analysis. The statistical test applied was differential gene expression using the RNA-seq package with CLC Genomics Workbench^©^ v 23.0 (Qiagen). The parameters for RNA-seq analysis were: strand setting – both, library type setting – bulk end Expression calculation for non-transcribed genes – yes. Library size was normalized using the trimmed mean of M-values (TMM) method (Robinson and Oshlack [16]). After calculating the TMM normalization for each sample, TMM-adjusted log c.p.m. counts were estimated (Robinson and Oshlack [16]). For mapping settings, the software used the Expectation–Maximization algorithm. Differential expression was assessed using multifactor statistics based on a negative binomial generalized linear model. The test used an adjusted false discovery rate (FDR) *P*-value. The parameters used to select the differentially expressed transcripts were a twofold change (FC >2 or <-2) and an FDR<0.05. The RNA-seq data are available at Sequence Read Archive, under the accession number PRJNA1476336 (SRR39062181, SRR39062182, SRR39062179, SRR39062183, SRR39062180 and SRR39062178).

#### Gene ontology analysis

To infer functional roles of differentially expressed transcripts, gene ontology (GO) analysis was performed in the Fungifun database (https://fungifun3.hki-jena.de/). Using the parameters: species – *C. neoformans* var. grubii H99; classification ontology: GO; gene IDs were inserted; significance test – hypergeometric distribution, test gene for category associations – over-representation; adjustment method – Benjamini–Hochberg procedure; annotation type – use only directed annotated associations; GO advanced options – all; filter by evidence code – select all evidence codes.

### Proteomics

#### Cell lysis

A 100 ml inoculum of liquid YPD medium was prepared in a sterile glass vial and incubated overnight at 30 or 37 °C under agitation. The following day, a cell count was performed for each inoculum. The samples were then centrifuged for 10 min at 5,000 ***g***, washed in sterile PBS and centrifuged again for 5 min at 5,000 ***g***. The cell pellet was suspended in PBS containing protease inhibitors, placed in 15 ml Falcon tubes, frozen on ice and then transferred to dry ice before being placed in the lyophilization equipment for ~4 h at 0.04 mbar and −50 °C. After lyophilization, a volume of glass beads was added, and 10 cycles of 1 min of vortexing followed by 1 min on ice were performed.

#### SDS-PAGE gel

The gel for protein resolution was prepared with 10% acrylamide (Bis/acrylamide (0.9/33%), Tris/HCl 2.5 M pH 8.8, SDS 10%, ammonium persulphate 10%, Tetramethylethylenediamine [TEMED]), applied to the plate and covered with Milli-Q water. After polymerization, the water was removed and the packing gel was applied. After polymerization, the system was washed with Milli-Q water and transferred to the electrophoresis apparatus containing running buffer. The samples were applied to the columns, and the run was performed at 120 V until the samples passed ~4 cm through the resolving gel.

#### Coomassie staining

The resolving gel containing the proteins extracted from *C. neoformans* cells was placed in Coomassie blue solution (0.1% Coomassie blue R-250, 45% methanol, 10% acetic acid) for 5 min. It was then placed in destaining solution (4% methanol, 7.5% acetic acid) overnight. The following day, the gel was washed three times with distilled water and stored in water until analysis.

#### In-gel protein digestion

The bands of each sample were excised from the gel with a scalpel and placed in decolourization buffer (sodium thiosulfate 100 mmol l^−1^ and potassium ferricyanide 30 mmol l^−1^). The tubes were placed in a thermomixer at 25 °C with shaking at 800 r.p.m. until the gel colour disappeared and a yellow colour appeared in the tube. Then, the supernatant was discarded, and the gel pieces were washed in the ABC solution (ammonium bicarbonate, 50 mmol l^−1^). The samples were again placed in the thermomixer at 25 °C with shaking at 800 r.p.m. until the gel was completely decolourized.

#### Reduction, alkylation and enzymatic digestion of proteins

For dehydration, the gel was placed in tubes containing 100% ethanol sufficient to cover it, which were then shaken in a thermomixer for 10 min at 25 °C (800 r.p.m.). The liquid was discarded, and the dehydration procedure was repeated until the gel turned white and hardened. Then, the gel was dried in a SpeedVac for 7 min at a vacuum pressure of 0.1; the reduction buffer [DTT (10 mmol l^−1^) in ABC (50 mmol l^−1^)] was added, and the mixture was incubated in the thermomixer for 60 min at 56 °C and 800 r.p.m.; the supernatant was discarded. Then, alkylation buffer [iodoacetamide solution (55 mmol l^−1^) in ABC (50 mmol l^−1^)] was added for another incubation protected from light for 45 min at 25 °C (800 r.p.m.), and the supernatant was discarded again. Digestion buffer (50 mmol l^-1^ ABC solution) was added to the tubes, which were incubated in a thermomixer for 20 min at 25 °C (800 r.p.m.); the supernatant was discarded. The samples were again dehydrated with 100% ethanol as previously described, and then the digestion buffer was added to the tubes. The samples were dehydrated twice more (or until the gel hardened and turned white) and then dried in the SpeedVac for 7 min. Finally, a 12.5 ng µ^l-1^ trypsin solution diluted in 50 mmol ^l-1^ ABC solution was added. The excess trypsin was discarded before adding digestion buffer, followed by incubation at 37 °C for 16–18 h.

#### Peptide extraction

The liquid from the tubes was collected after incubation at 37 °C, and the extraction solution (water, 3% TFA, 30% acetonitrile) was added. The tubes were incubated in a thermomixer for 10 min at 25 °C (800 r.p.m.), and the supernatant was collected. The procedure was repeated. Then, acetonitrile was added to the tubes, and after shaking in the thermomixer, the supernatant was transferred to a new tube. The procedure was repeated. The samples were dried in the SpeedVac to 10–20% of the initial volume, and the peptides were purified using StageTips-C18.

#### Mass spectrometry

The sample analysis was performed on the Ultimate 3000 RSLCnano liquid chromatograph (Thermo Scientific) on the RPT02H platform (Carlos Chagas Institute, Fiocruz - PR). A gradient (Phase A: 0.1% formic acid; Phase B: 0.1% formic acid, 95% acetonitrile) was performed at a flow rate of 250 nl min^−1^. A linear gradient from 5 to 40% acetonitrile was applied over 120 min using a 15 cm long analytical column with an internal diameter of 75 µm containing C18 particles with a diameter of 3 µm. Spectrometry was performed using an Orbitrap Fusion Lumos (Thermo Scientific). For electrospray ionization, the source parameters were set to 2,300 V and 175 °C, the detector was set to a resolution of 120,000 using EASY-IC (internal calibration), the mass range was 300–1,500 m/z, the threshold intensity was 2×10^4^ and the exclusion duration was 60 s.

#### Protein analysis

The data generated by MaxQuant (v.1.5.5.1) were analysed using Perseus (v.1.6.14.0), where proteins considered potential contaminants, proteins with reverse sequences and proteins identified by only one site were first removed. Similar samples were treated as replicates and proteins were filtered to retain only those detected in at least two of the three replicates. No additional imputation of missing values was performed. Gene annotations were performed using genome annotations from the JEC21 strain available in the Perseus annotations database. The Student’s t-test was applied with an FDR of 0.05 or less and the resulting data were filtered for a fold change greater than 1.5 or less than −1.5. The proteins that passed the filters were selected for analysis.

#### GO analysis

The GO analysis was performed in the Fungifun database (https://fungifun3.hki-jena.de/). Using the parameters: species – *C. neoformans* var. *neoformans* serotype D (strain JEC21); classification ontology: GO; gene IDs were inserted; significance test – hypergeometric distribution, test gene for category associations – over-representation; adjustment method – Benjamini–Hochberg procedure; annotation type – use only directed annotated associations; GO advanced options – all; filter by evidence code – select all evidence codes.

#### Transcriptomic–proteomic integration and correlation analysis

To assess the concordance between transcriptomic and proteomic responses to growth at 37 °C, transcript and protein datasets were integrated based on the corresponding *C. neoformans* H99 gene identifiers (CNAG IDs). For each matched feature, the log_2_ fold change (37 versus 30 °C) obtained from the RNA-seq analysis was paired with the corresponding protein log_2_ fold change from the proteomic dataset. Features without a corresponding identifier in the other dataset were excluded from the paired analysis.

The relationship between transcript and protein fold changes was assessed using both Pearson’s correlation coefficient (r) and Spearman’s rank correlation coefficient (ρ). Statistical significance was assessed using a two-sided test, with *P*<0.05 considered statistically significant.

To further characterize concordant responses, matched features were classified according to the direction of their changes at the transcript and protein levels. Features showing increased abundance at both levels or decreased abundance at both levels were considered directionally concordant, whereas features showing opposite changes were considered discordant. For the functional analysis, concordantly regulated features that met the significance criteria in both datasets were subjected to GO enrichment analysis separately according to the direction of regulation. GO enrichment was assessed using the same statistical framework and multiple-testing correction described above.

#### XTT assay

H99 cells were grown at 30 and 37 °C for 24 h. The following day, inocula of 5×10^7^ cells ml^−1^ were washed 3× with PBS and finally diluted in minimal media in a 96-well plate (five biological replicates per condition) for incubation for 20 h at 30 or 37 °C. After the incubation period, 1 mg ml^−1^ of XTT (Sigma-Aldrich) and 1 mM of Menadione in acetone (Sigma-Aldrich) were added to each well, and the plates were incubated at the same temperatures for an additional 4 h. The plates were then centrifuged at 750 ***g*** for 1 min, and the supernatant transferred to new wells for absorbance measurement at 490 nm using a microplate reader (Synergy H1, Biotek). XTT reduction at 30 and 37 °C was compared using a two-tailed unpaired Student’s t-test.

## Results

### Differential gene expression

To characterize the transcriptional response associated with growth at environmental and host-associated temperatures, we analysed the transcriptome of the *C. neoformans* H99 strain after 24 h of growth at 30 and 37 °C. Unlike previous studies comparing growth at 25 and 37 °C [4], our experimental design focused on comparing an environmental temperature with mammalian physiological temperature. We identified 772 differentially expressed transcripts using a threshold of |log_2_ fold change|≥1 and FDR ≤5%, including 344 upregulated and 428 downregulated transcripts at 37 °C compared with 30 °C (Fig. 1 and Table S1, available in the online Supplementary Material).

**Fig. 1.**
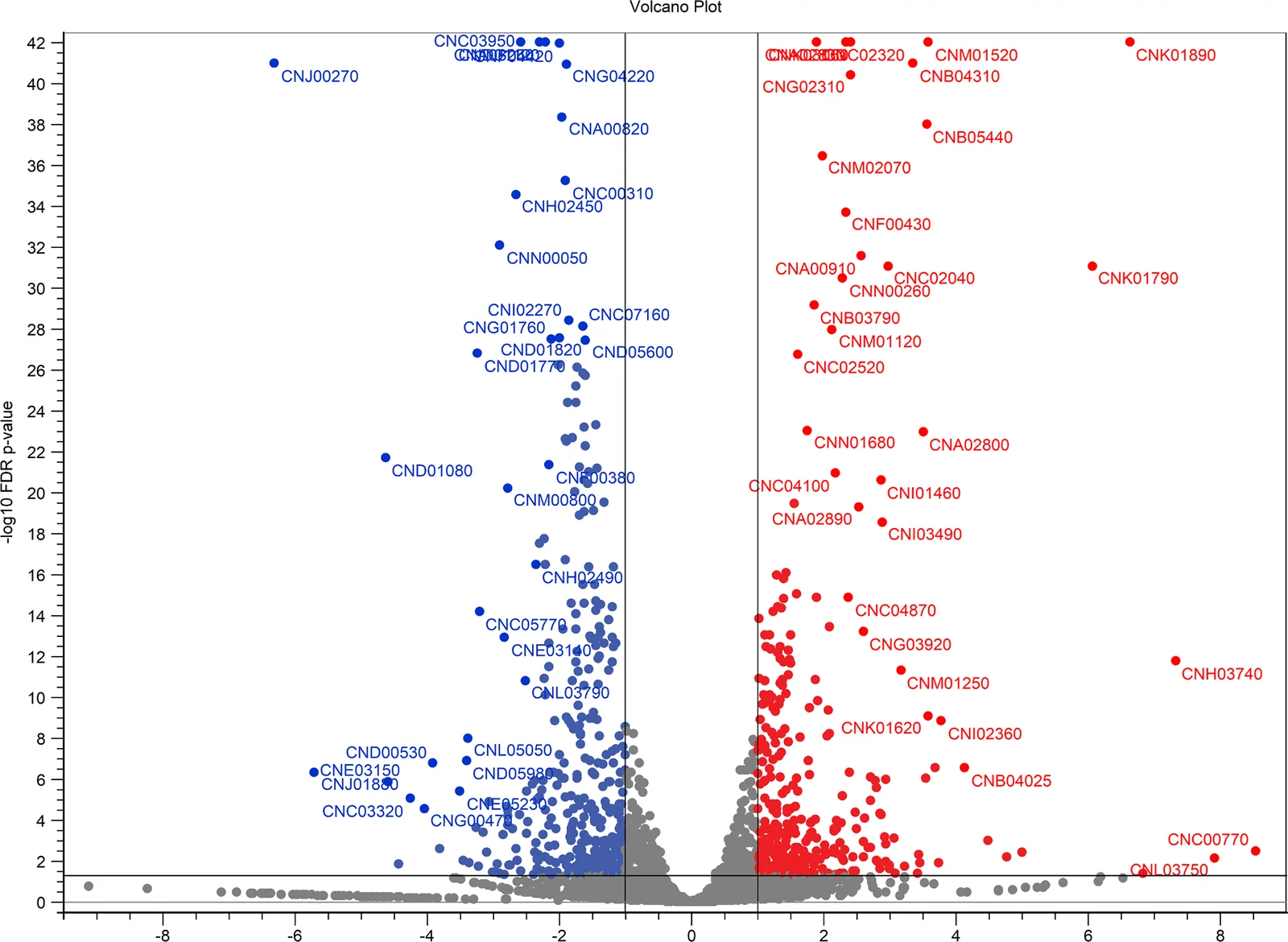
Volcano plot of the transcripts identified in this analysis, comparing the H99 samples grown at 30 and 37 °C. The log_2_ fold change values (X axis) are shown, with red points corresponding to gene transcripts that are upregulated and blue points corresponding to those that are downregulated in the samples grown at 37 °C. The Y axis represents the −log_10_ FDR statistical value (corrected *P*-value). The grey points correspond to the transcripts that did not pass the statistical criterion.

Among the upregulated transcripts, several functional categories were associated with protein homeostasis, translation, RNA-associated functions, ATP binding, oxidoreductase activity and electron transport. Four rRNA transcripts (CNAG_10503, CNAG_10501, CNAG_10502, and CNAG_10500) and one ncRNA (CNAG_13073) were also significantly upregulated at 37 °C. The increased representation of ribosome- and translation-associated transcripts is consistent with previous studies describing changes in protein synthesis and proteostasis during adaptation to elevated temperature [17].

GO enrichment analysis of the upregulated transcripts identified categories related to rRNA binding (58.33%), NADH dehydrogenase activity (42.86%), unfolded protein binding (39.19%), translation (32.23%), protein folding (31.43%), ribosome (30.08%) and structural constituents of the ribosome (29.69%) (Fig. 2). These results indicate that growth at 37 °C is associated with increased representation of transcriptional functions related to ribosome organization, protein synthesis and protein quality control.

**Fig. 2.**
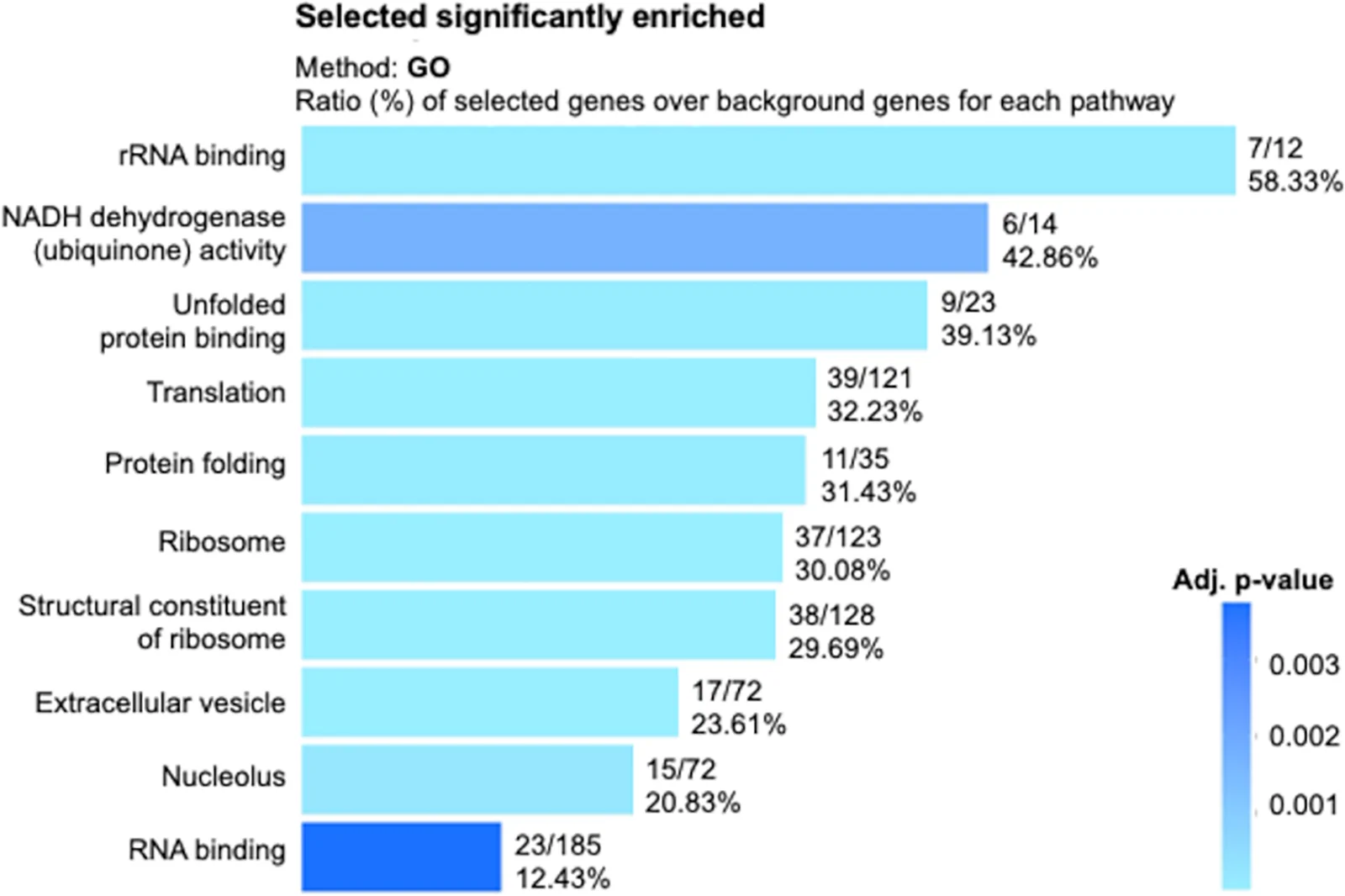
GO plot of enriched terms of the upregulated genes of the strain H99 grown at 37 °C. Image created using FungiFun3.

Previous studies have shown that the transcriptional response to elevated temperature is dynamic. For example, transcripts encoding ribosomal proteins undergo transient reduction during the first 3 h following temperature elevation [2], whereas infection-mimicking conditions have been associated with increased ribosome biogenesis after 6 h at host temperature [18]. The increased representation of ribosome-associated and protein-folding functions observed after 24 h in our study is therefore consistent with a later stage of adaptation to growth at 37 °C rather than an acute heat-shock response.

The upregulated transcript set also included categories associated with mitochondrial and redox-related functions, including NADH dehydrogenase activity, ATP binding, oxidoreductase activity and electron transport. These findings indicate that elevated temperature is accompanied by transcriptional changes in cellular functions associated with energy metabolism and redox processes. However, these transcriptomic changes do not by themselves establish increased mitochondrial respiratory capacity or ATP production.

In contrast, downregulated transcripts were enriched in categories associated with carbon–sulphur lyase activity, phosphatidylserine decarboxylation, nitrogen utilization, oxidoreductase functions and carbohydrate metabolism (Fig. 3). These results indicate that growth at 37 °C is also associated with reduced representation of selected metabolic and biosynthetic functions. The observed changes may reflect differences in the cellular requirements associated with growth at the two temperatures; however, the transcriptomic data alone do not establish a redistribution of metabolic resources or changes in metabolic flux.

**Fig. 3.**
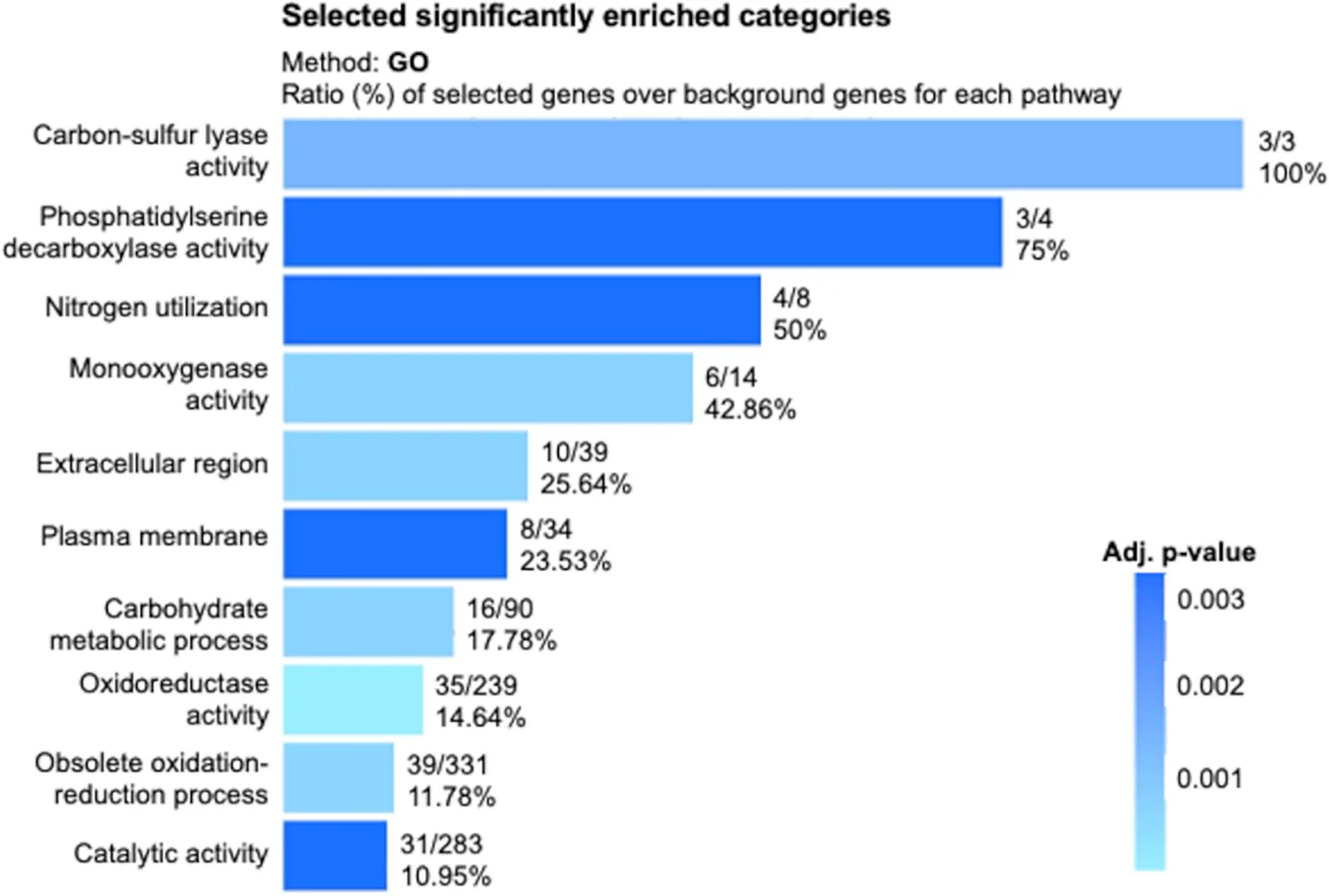
GO plot of terms enriched in the downregulated genes of the strain H99 grown at 37 °C. Image created using FungiFun3.

Overall, the transcriptomic response to 37 °C was characterized by broad remodelling of cellular functions, with increased representation of ribosome-associated, translation, protein-folding, mitochondrial-associated and redox-related processes, accompanied by changes in selected carbohydrate, nitrogen, lipid and other metabolic functions. These findings indicate that growth at host temperature is associated with substantial transcriptional remodelling involving protein synthesis, proteostasis and metabolism.

### Proteomic profile

To determine whether the transcriptional changes observed during thermal adaptation were reflected at the protein level, we performed a comparative proteomic analysis of *C. neoformans* cells grown at 30 and 37 °C. A total of 2,970 proteins were initially identified. After removing contaminants, reverse sequences and proteins detected by a single peptide, proteins with at least two valid measurements per experimental group were retained, resulting in a final dataset of 1,901 proteins for downstream analyses.

Differential protein abundance was assessed using Student’s t-test (FDR<0.05) combined with a fold-change threshold of ≥1.5. Despite the extensive transcriptional remodelling observed at 37 °C, 177 proteins met the statistical criteria (Table S2). The proteins showing the largest changes in abundance are presented in Table 1.

**Table 1.** Differentially abundant proteins identified in *C. neoformans* H99 cells grown at 37 °C (blue) compared with 30 °C (orange). The table shows the H99 strain gene identifier, protein description, corresponding JEC21 strain identifier, log_2_ fold change (37/30 °C) and FDR. Positive log_2_ fold-change values indicate increased protein abundance at 37 °C, whereas negative values indicate decreased abundance. Proteins are presented according to their differential abundance, with FDR values ranging from 0 to 5%

H99 strain ID	Description	JEC21 strain ID	Log_2_ FC	FDR (%)	Temperature (°C)
CNAG_07163	Preprotein translocase subunit YidC	CNE02790	1.81	1	37 °C
CNAG_01464	Flavohaemoglobin	CNC07160	1.60	2	
CNAG_00730	ATP-binding cassette transporter	CNA07090	1.64	2	
CNAG_04862	Glutamate synthase (NADPH/NADH), variant	CNJ02910	1.68	5	
CNAG_06453	Benzodiazepine receptor	CNN01770	−1.85	5	30 °C
CNAG_03572	Opsin 1	CNG00350	−1.63	4	
CNAG_05721	Multifunctional β-oxidation protein	CNF02530	−1.98	2	
CNAG_00247	α-Aminoadipic semialdehyde synthase	CNA02370	−2.26	1	
CNAG_05229	Stomatin family protein	CNL03990	−1.73	1	
CNAG_02489	Alcohol dehydrogenase, propanol-preferring	CNE00710	−3.28	1	
CNAG_06431	Acyl-CoA oxidase	CNN01550	−2.45	1	
CNAG_06294	Hypothetical protein	CNN00300	−5.14	5	
CNAG_06628	Aldehyde dehydrogenase (NAD)	CNF03900	−5.73	0	

GO enrichment analysis was performed to further characterize the differentially abundant proteins identified at 37 °C. Among the proteins showing increased abundance, significantly enriched categories included oxidoreductase activity, hydroxymethylglutaryl-CoA synthase activity, sterol biosynthetic process, glutamine metabolic process, isoprenoid biosynthetic process, haem binding and iron ion binding (Fig. 4). The broader categories catalytic activity, oxidation-reduction process and oxidoreductase activity were also significantly enriched.

**Fig. 4.**
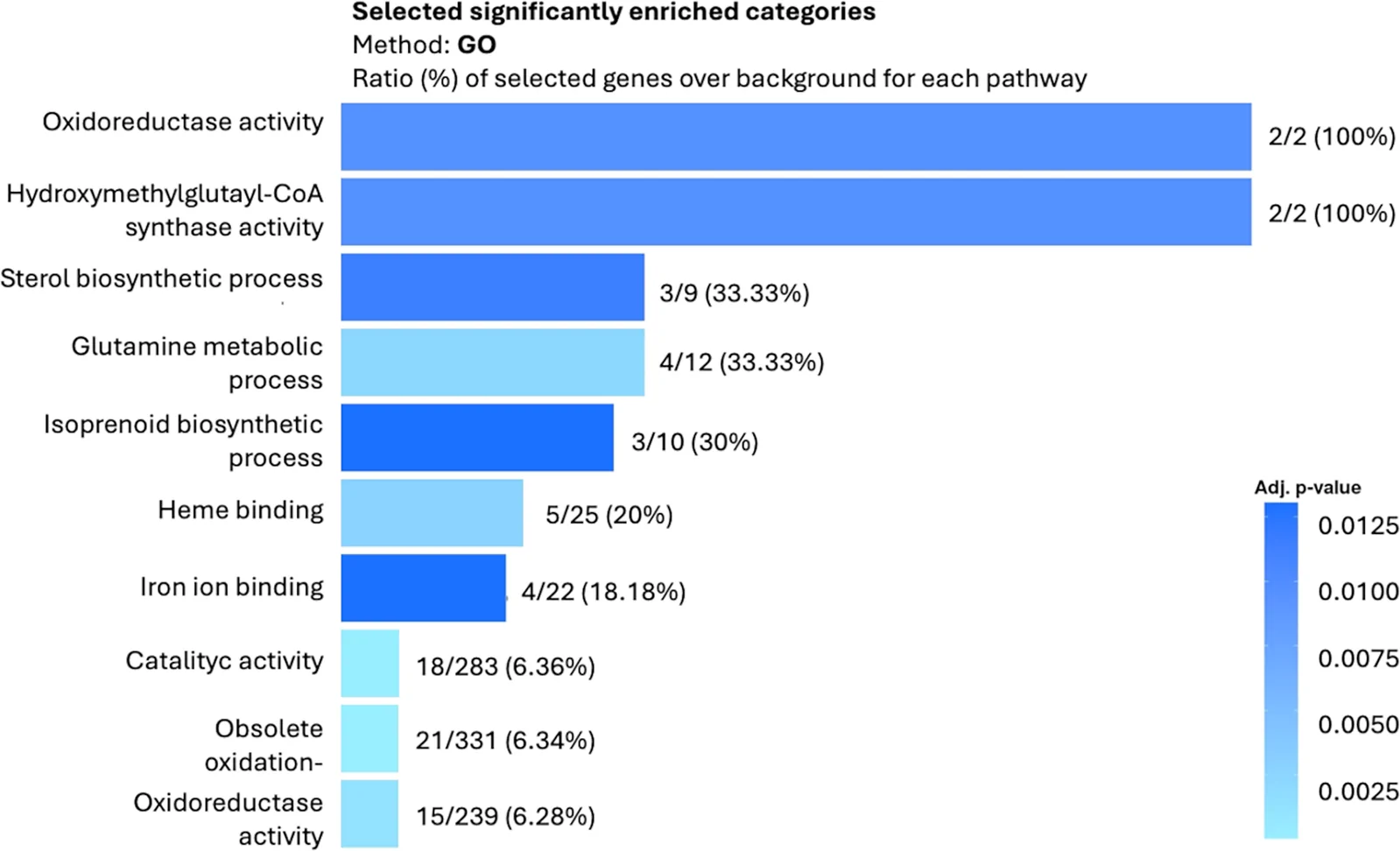
GO term analysis of proteins increased in cells of the H99 strain grown at 37 °C versus 30 °C, which passed the statistical filter (t-test difference >1.5). Image created using FungiFun3.

In contrast, no GO categories reached statistical significance among the proteins showing decreased abundance at 37 °C. Thus, the functional enrichment analysis provided evidence for selective enrichment of specific metabolic, biosynthetic and redox-related functions among the proteins increased at 37 °C, whereas no significantly enriched functional category could be identified among the proteins with decreased abundance.

### Transcriptomic–proteomic concordance and functional enrichment

To further assess the relationship between the transcriptomic and proteomic responses, we compared the log_2_ fold changes of 305 matched transcript–protein pairs. Overall, 162 features (53.1%) showed concordant changes between the two datasets, including 61 that increased and 101 that decreased at both the transcript and protein levels. In contrast, 143 features showed changes in opposite directions (Fig. 5) (Table S3).

**Fig. 5.**
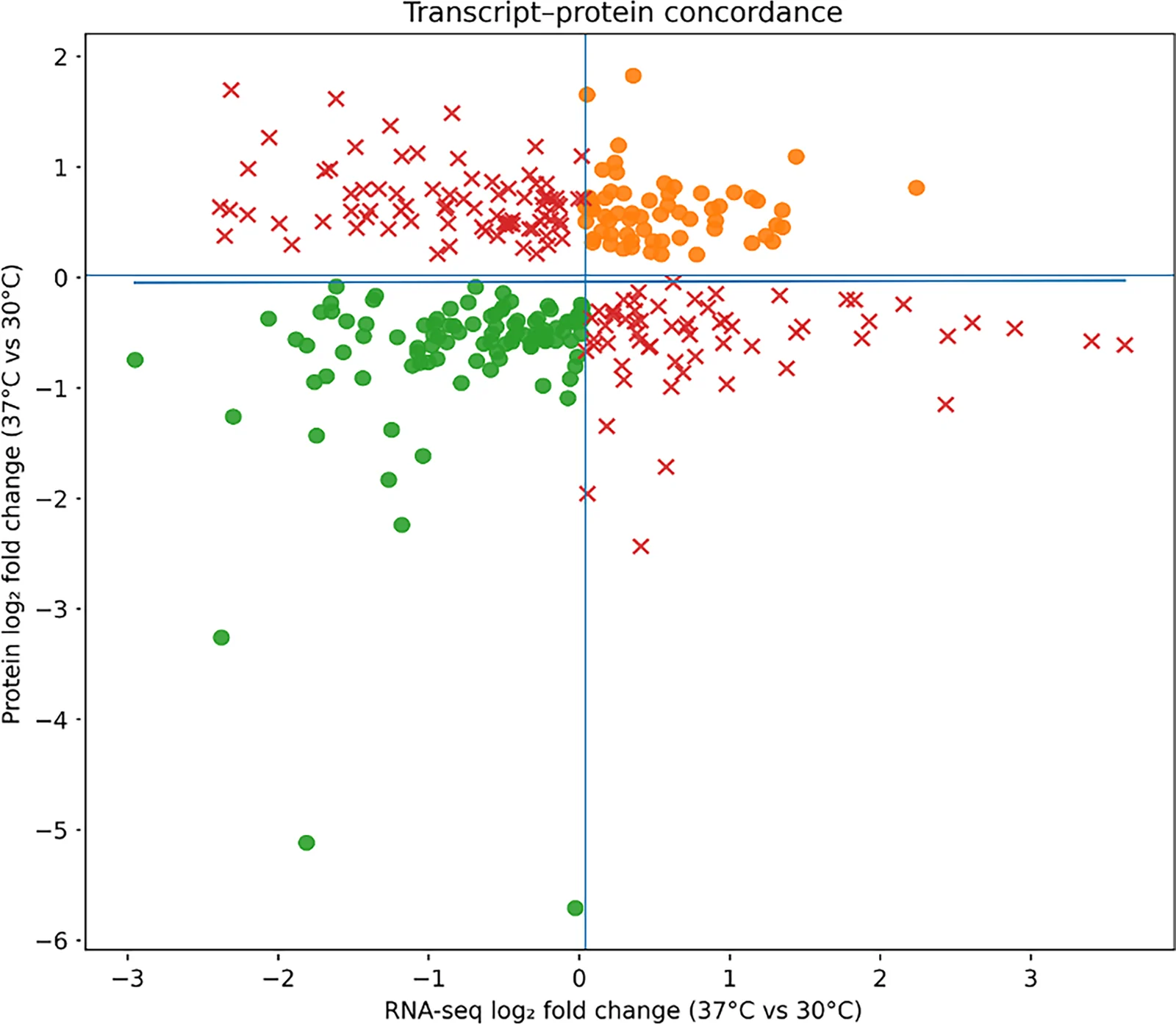
Transcript–protein concordance analysis. Scatter plot between RNA-seq and protein log_2_ fold changes comparing *C. neoformans* H99 cells grown at 37 °C versus 30 °C. Each point represents a matched transcript–protein pair. Orange, concordantly upregulated features (RNA ↑/protein ↑; *n*=61); green, concordantly downregulated features (RNA ↓/protein ↓; *n*=101); and blue crosses, discordant data (*n*=143). X-axis: Transcript abundance, Y-axis: Protein abundance (Spearman’s ρ = −0.005, *P*=0.934; Pearson’s *r*=0.003, *P*=0.959).

Among the concordantly increased proteins, several notable changes were observed. These included the preprotein translocase subunit YidC (CNAG_07163), the ABC transporter AFR1 (CNAG_00730), the F-type H^+^-transporting ATPase subunit G (CNAG_02762), NADH dehydrogenase (CNAG_05434), methylenetetrahydrofolate dehydrogenase (CNAG_07746), mitochondrial aconitate hydratase (CNAG_07908), HMG-CoA synthase (ERG13; CNAG_03311) and catalase 2 (CAT2; CNAG_05256). These proteins showed increased abundance at 37 °C together with increased transcript levels, providing direct evidence of concordant regulation at both molecular levels.

Among the concordantly decreased proteins, the most pronounced changes included aldehyde dehydrogenase (CNAG_06628), alcohol dehydrogenase (CNAG_02489), LYS9 (CNAG_00247), CAT1 (CNAG_04981), methylmalonate-semialdehyde dehydrogenase (CNAG_04351), fumarate reductase (CNAG_07862), malate synthase A (MLS1; CNAG_05653), acetyl-CoA acyltransferase (CNAG_00490), glutathione S-transferase Gst3 (CNAG_01893) and NADH dehydrogenase (CNAG_00254). These proteins showed reduced abundance together with reduced transcript levels at 37 °C.

Overall, the concordant changes identified at both transcript and protein levels highlight specific proteins involved in cellular metabolism, redox homeostasis, protein transport and mitochondrial-associated functions that respond consistently to growth at 37 °C.

### Metabolic activity enrichment at higher temperatures

Consistent with the molecular changes identified in the transcriptomic and proteomic datasets, we next assessed cellular metabolic activity using the XTT reduction assay [17–19]. XTT reduction is based on the conversion of the tetrazolium salt XTT to a coloured formazan product by cellular reducing activity, with the reaction enhanced by the electron-coupling agent menadione [20]. Because XTT reduction can be influenced by cellular metabolic and redox processes, this assay was used here as an indirect measure of cellular metabolic activity rather than as a direct measurement of mitochondrial respiration or oxidative phosphorylation.

As shown in Fig. 6, background absorbance from XTT alone and fungal cells alone was negligible at both temperatures. In contrast, cells incubated with XTT showed a clear increase in absorbance, consistent with active reduction of the substrate. Cultures grown at 37 °C displayed significantly greater XTT reduction than cultures grown at 30 °C (*P*=0.01), despite being standardized to the same cell density. Under these experimental conditions, the higher XTT signal therefore indicates increased cellular metabolic activity at 37 °C.

**Fig. 6.**
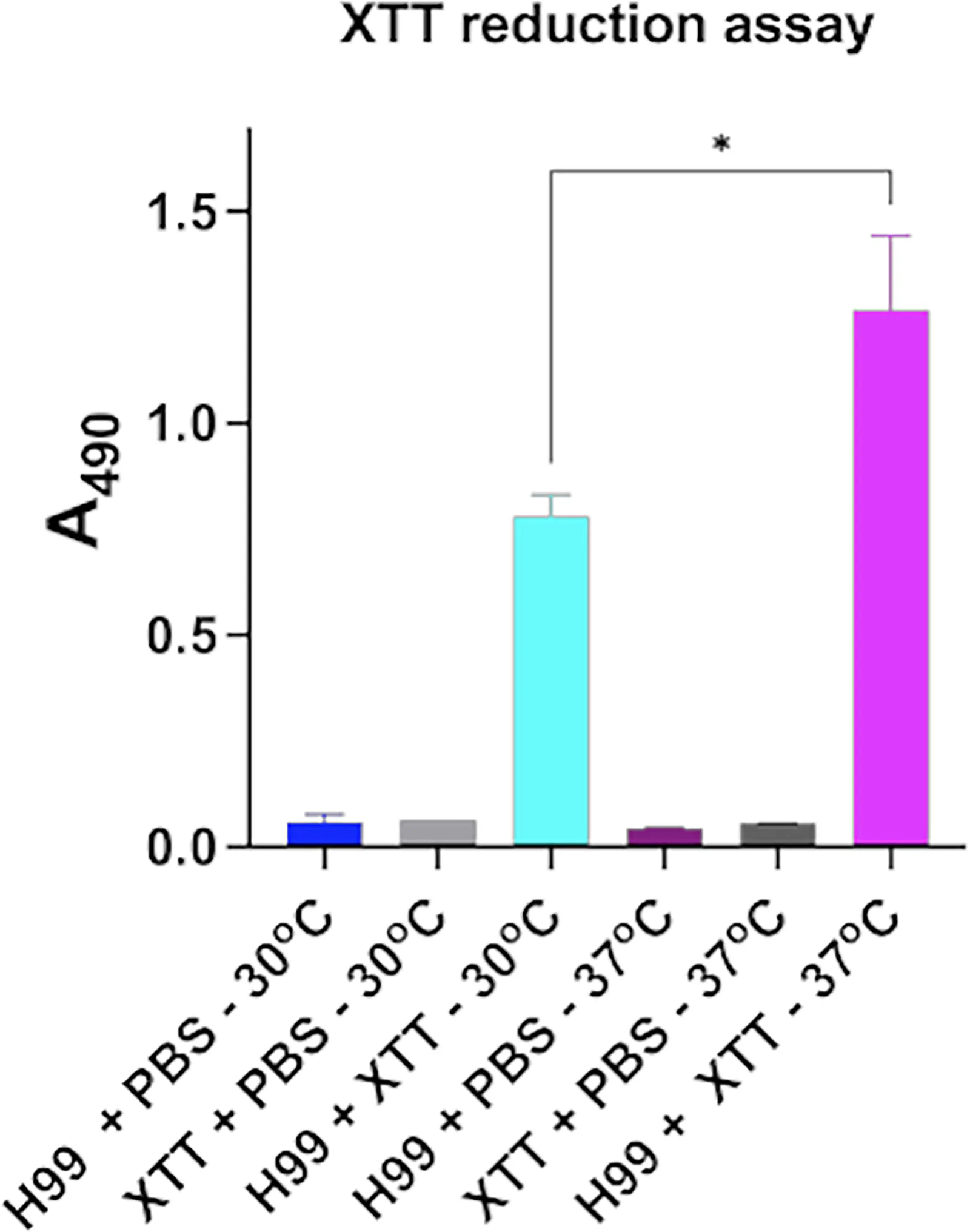
XTT reduction assay. The graph shows the absorbance at 490 nm for each condition tested: negative control (H99+PBS), background control (XTT+PBS) and the test sample (H99+XTT), at two temperatures: 30 and 37 °C. A two-tailed unpaired Student’s t-test was used to compare H99+XTT at 30 and 37 °C (*P*=0.01).

Overall, the XTT results provide an independent functional measure of the metabolic differences observed between cells grown at 30 and 37 °C. The increased XTT reduction at 37 °C is consistent with the metabolic changes identified by the transcriptomic and proteomic analyses. However, because XTT reduction is an indirect assay, these results do not by themselves establish increased mitochondrial respiratory capacity, oxidative phosphorylation or metabolic flux.

## Discussion

Adaptation to mammalian body temperature is an important prerequisite for the pathogenic lifestyle of *C. neoformans[21]*. In this study, integrated transcriptomic, proteomic and functional analyses showed that growth at 37 °C is associated with broad molecular remodelling. The transcriptomic response was characterized by changes in processes related to translation, ribosome biogenesis, protein folding and metabolism, whereas the proteomic response was considerably more selective. Direct comparison of transcript and protein fold changes revealed no significant quantitative correlation, although 53.1% of matched features showed concordant directional changes. These findings indicate that the response to host temperature involves regulation at multiple molecular levels and that transcript abundance is not uniformly reflected at the protein level.

Previous studies have established that *C. neoformans* grows more efficiently at environmental temperatures near 30 °C than at the 37 °C encountered in mammalian hosts [22]. Nevertheless, the fungus can adapt to host temperature through extensive changes in gene expression and cellular physiology. Early transcriptomic analyses of the H99 strain reported increased expression of genes involved in translation and protein synthesis at elevated temperatures, including elongation factor-1α and multiple ribosomal proteins [4]. Our results are consistent with these observations. Among the transcript categories enriched at 37 °C were ribosome-associated functions, including rRNA binding, structural constituents of the ribosome, translation and protein folding. The increased representation of rRNA-related transcripts is also consistent with changes in ribosome biogenesis and translational functions during adaptation to host temperature. Ribosome production is an energetically demanding cellular process that requires coordinated regulation of rRNA synthesis, ribosomal protein expression and assembly factors [23]. The enrichment of ribosome-related transcripts observed here may therefore reflect changes in the cellular requirements for protein synthesis and proteostasis during prolonged exposure to 37 °C. This interpretation is consistent with previous reports describing dynamic regulation of ribosomal genes during environmental adaptation, including transient repression during acute stress followed by induction during sustained adaptation and recovery [2, 24].

Mitochondrial and metabolic functions were also represented among the molecular changes detected at 37 °C, although the extent of these changes differed between the transcriptomic and proteomic datasets. The transcriptomic analysis identified changes in genes associated with NADH dehydrogenase activity, ATP binding and components of the respiratory electron transport chain. In the proteomic dataset, proteins increased at 37 °C were associated with oxidoreductase activity, dehydrogenase functions, tetrapyrrole biosynthesis and other metabolic processes. However, the limited quantitative concordance between transcript and protein abundance indicates that these datasets should not be interpreted as equivalent measures of the same response. Instead, they provide complementary information on distinct levels of cellular regulation. Consistent with this view, GO analysis of the concordantly regulated transcript–protein subset identified specific metabolic and biosynthetic categories among increased features and respiratory, proton transport and redox-related categories among decreased features.

These findings are consistent with previous studies identifying mitochondrial functions as components of the response of *C. neoformans* to elevated temperature [25–28]. One of the highly induced transcripts in our dataset encoded a mitochondrial NADH dehydrogenase, a protein previously associated with respiratory activity and virulence in *C. neoformans* [17]. However, the present data do not directly establish increased mitochondrial respiratory capacity. Rather, they identify changes in transcripts and proteins associated with mitochondrial and metabolic functions during growth at 37 °C. This distinction is particularly relevant because the XTT assay used in this study provides an indirect measure of cellular metabolic activity rather than a direct measurement of mitochondrial respiration or oxidative phosphorylation.

The XTT assay nevertheless provided an independent functional measurement of cellular metabolic activity under the experimental conditions used. Cells grown at 37 °C showed significantly greater XTT reduction than cells grown at 30 °C despite normalization to the same cell density. This result is consistent with differences in cellular metabolic activity between the two temperature conditions and provides functional context for the molecular changes identified by the omics analyses. However, XTT reduction can reflect multiple cellular reducing processes and should therefore not be interpreted as direct evidence of increased respiratory capacity or metabolic flux.

The proteomic dataset also identified changes in proteins associated with fatty acid and aa metabolism, including acyl-CoA oxidase, β-oxidation-associated proteins and saccharopine dehydrogenase. These changes indicate that pathways involved in carbon and nitrogen metabolism are affected by growth at 37 °C, although their functional consequences cannot be inferred directly from the present dataset. These observations indicate that multiple metabolic functions are affected by growth at 37 °C. Importantly, the absence of a strong quantitative correlation between transcript and protein fold changes suggests that these responses are not uniformly coupled across molecular levels. The functional enrichment observed among the concordant features therefore provides a more restricted view of the processes that show consistent regulation at both levels.

We also observed enrichment of extracellular vesicle-related transcripts at 37 °C. Extracellular vesicles are involved in the transport of proteins, lipids, RNA and other molecules [29], and previous studies have reported changes in their composition under stress conditions in *C. neoformans* [30, 31]. Our results suggest that vesicle-associated functions may be affected by growth at 37 °C. However, further experiments are needed to determine whether these changes affect extracellular vesicle production or composition.

These findings also have relevance to the immunopathogenesis of cryptococcosis because the physiological state adopted by *C. neoformans* at host temperature may influence its interaction with host immune cells. Mitochondrial and metabolic remodelling has been linked to the physiological behaviour of *C. neoformans* during macrophage infection [32].

Interestingly, only a subset of genes previously implicated in thermotolerance showed increased expression under our experimental conditions. Transcripts of *TSA1* and *ILV2* were increased at 37 °C, whereas *CNB1*, *SOD2*, *TPS1*, *TPS2* and *LYS9* were decreased. This apparent difference from previous studies may reflect differences in experimental design and biological context. Many thermotolerance-associated genes were identified through mutant phenotyping, which establishes a requirement for a gene in a particular phenotype but does not necessarily predict its expression pattern during adaptation. In addition, our analyses were performed after 24 h of growth at 37 °C and therefore likely represent a later stage of adaptation rather than an acute heat-shock response. The transcriptional changes observed here should consequently be interpreted in the context of prolonged exposure to host temperature.

A limitation of the present study is that cells were compared after a fixed 24 h incubation rather than at matched physiological or growth phases. Therefore, some of the observed differences may reflect temperature-dependent differences in growth kinetics or cellular state in addition to temperature-specific regulatory responses. Future studies using matched growth phases and multiple time points will be important to distinguish these effects from the molecular response specifically associated with thermal adaptation.

Taken together, our results indicate that exposure of *C. neoformans* to 37 °C is associated with broad transcriptional remodelling and a more selective proteomic response. The two molecular layers showed limited overall quantitative concordance, but the subset of concordantly regulated features was enriched in specific metabolic, biosynthetic, respiratory and redox-related functions. Increased cellular metabolic activity measured by XTT further demonstrates a functional difference between cells grown at 30 and 37 °C, although this assay does not directly establish changes in mitochondrial respiration. Overall, these findings support a model in which adaptation to host temperature involves coordinated changes in protein synthesis, proteostasis and selected metabolic and cellular functions. Such remodelling may contribute to the ability of *C. neoformans* to maintain cellular homeostasis under host-temperature conditions, while further functional studies will be required to determine how these molecular changes contribute to pathogenicity.

## Conclusions

Growth of *C. neoformans* H99 at 37 °C was associated with broad transcriptional remodelling and a more selective proteomic response, particularly involving translation, protein homeostasis and metabolic functions. Transcript–protein integration revealed 53.1% directional concordance among matched features, but no significant quantitative correlation between transcript and protein fold changes. Concordantly regulated features were enriched in specific metabolic, biosynthetic, respiratory and redox-related functions. Increased XTT reduction further demonstrated higher cellular metabolic activity at 37 °C under the conditions tested. Together, these findings identify coordinated cellular processes associated with growth at host temperature and provide a framework for future studies investigating their contribution to thermoadaptation and cryptococcal pathogenicity.

## Supplementary material

10.1099/jmm.0.002205 Supplementary Material 1.

## Abbreviations

ABC: ammonium bicarbonate
FDR: false discovery rate
GO: gene ontology
SAGE: Serial Analysis of Gene Expression
TEMED: tetramethylethylenediamine
TMM: trimmed mean of M-values
XTT: 2,3-bis-(2-methoxy-4-nitro-5-sulfophenyl)-2H-tetrazolium-5-carboxanilide
YPD: yeast extract peptone dextrose
